# CACSNet for automatic robust classification and segmentation of carotid artery calcification on panoramic radiographs using a cascaded deep learning network

**DOI:** 10.1038/s41598-024-64265-4

**Published:** 2024-06-17

**Authors:** Suh-Woo Yoo, Su Yang, Jo-Eun Kim, Kyung-Hoe Huh, Sam-Sun Lee, Min-Suk Heo, Won-Jin Yi

**Affiliations:** 1 Department of Oral and Maxillofacial Radiology, School of Dentistry, Seoul National University, Seoul, Korea; 2 Department of Applied Bioengineering, Graduate School of Convergence Science and Technology, Seoul National University, Seoul, Korea; 3 Department of Oral and Maxillofacial Radiology and Dental Research Institute, School of Dentistry, Seoul National University, Seoul, Korea

**Keywords:** Carotid artery calcification, Panoramic radiograph, Deep learning network, Classification, Segmentation, Panoramic radiography, Biomedical engineering, Machine learning, Stroke

## Abstract

Stroke is one of the major causes of death worldwide, and is closely associated with atherosclerosis of the carotid artery. Panoramic radiographs (PRs) are routinely used in dental practice, and can be used to visualize carotid artery calcification (CAC). The purpose of this study was to automatically and robustly classify and segment CACs with large variations in size, shape, and location, and those overlapping with anatomical structures based on deep learning analysis of PRs. We developed a cascaded deep learning network (CACSNet) consisting of classification and segmentation networks for CACs on PRs. This network was trained on ground truth data accurately determined with reference to CT images using the Tversky loss function with optimized weights by balancing between precision and recall. CACSNet with EfficientNet-B4 achieved an AUC of 0.996, accuracy of 0.985, sensitivity of 0.980, and specificity of 0.988 in classification for normal or abnormal PRs. Segmentation performances for CAC lesions were 0.595 for the Jaccard index, 0.722 for the Dice similarity coefficient, 0.749 for precision, and 0.756 for recall. Our network demonstrated superior classification performance to previous methods based on PRs, and had comparable segmentation performance to studies based on other imaging modalities. Therefore, CACSNet can be used for robust classification and segmentation of CAC lesions that are morphologically variable and overlap with surrounding structures over the entire posterior inferior region of the mandibular angle on PRs.

## Introduction

The second most common cause of death worldwide is stroke, which is responsible for 11% of total deaths according to the WHO^1^. Strokes can be divided into hemorrhagic and ischemic strokes. Hemorrhagic strokes are caused by ruptured arteries while ischemic strokes are caused by clots or other obstructions/occlusions within arteries^2^. Approximately 60% of all acute strokes are associated with atherosclerotic disease in the common carotid artery^3^. Atherosclerotic lesions are most commonly found at branch points and bifurcations of blood vessels. Carotid artery atherosclerosis typically affects the carotid bifurcation^4^. Several noninvasive imaging techniques are currently used to evaluate atherosclerotic carotid artery stenosis^5–9^, including MRI^7^, CT^8^, and Doppler ultrasound^9^. CT angiography is a robust technique for assessing calcification and quantifying atherosclerosis load in the carotid arteries^10^.

Panoramic radiography (PR) is a standard routine imaging technique in initial examination in dental clinics. Several studies have described the usefulness of PR as a screening tool to diagnose carotid artery calcification (CAC) and investigated the prevalence of CAC in various populations^11–14^. CAC evaluation by PRs could aid in the identification of patients at high risk of cerebrovascular accident^15^. However, distinguishing CAC on PRs from other calcified anatomic structures or pathoses is difficult, and subject to subjective interpretation^16^. Even dental professionals with appropriate training find it challenging to accurately diagnose CAC in PR as it requires considerable experience and expertise^17^. Consequently, conventional diagnosis of CAC from PRs has limited accuracy. However, the introduction of an automatic assistance method could mitigate inter-examiner variability and facilitate a more reliable and precise evaluation of CAC on PRs^18^.

Deep learning-based approaches have gained importance in medical image analysis tasks such as classification, detection, and segmentation^19^. To detect CAC on PRs, Kats et al. applied Faster R-CNN with data augmentation by flipping, rotating, and changing the brightness to overcome the small size of their dataset, and reported a sensitivity of 0.75 and specificity of 0.80^20^. The performance of InceptionResNetV2, DenseNet169, and EfficientNetV2M models for detecting CAC from PRs was compared, and transfer learning was used to address the small number of image samples; the best-performing model had a sensitivity of 0.82 and specificity of 0.97^21^. There have been several studies on the automatic segmentation of CAC from MR, CT, and ultrasound images using deep learning. A two-stage deep learning-based method was proposed for segmentation of carotid atherosclerotic plaques in multi-weighted MR images^22^. A deep learning-based method was developed to segment CAC in CT angiography^23^. A deep learning network combining U-Net and DenseNet was used to segment carotid plaques in ultrasound images^24^. Segmentation of the CACs has proven beneficial for quantitatively assessing cardiovascular disease risk by providing detailed information about the size, location, and shape of the CACs. However, to date, no studies have investigated automatic segmentation of CAC lesions on PRs using deep learning.

Providing accurate ground truth data is essential for obtaining high performance in deep learning models. However, accurate labeling of the ground truth for CAC lesions on 2D projection PRs poses a challenge in the automatic segmentation of CAC using deep learning. This challenge arises from the difficulty in distinguishing between CAC lesions and surrounding anatomical structures with precision, compounded by the large pathological variations exhibited by the lesions. Therefore, the purpose of this study was to automatically and robustly classify and segment the CACs overlapping with anatomical structures and showing large variations in size, shape, and location on PR using a cascaded deep learning network. The model consisted of cascaded deep learning networks of classification for normal or abnormal PRs, and segmentation of CAC lesions in abnormal images only to provide accurate and robust segmentation of CACs in PRs. Our main contributions are as follows. (1) We developed a cascaded deep learning network (CACSNet) for automatic robust classification and segmentation of CACs on PRs, trained on accurate ground truth data with reference to CT images. (2) We employed various backbones and the Tversky loss function with optimized weights by balancing between precision and recall to improve our network’s CAC segmentation performance.

## Materials and methods

### Data acquisition and preparation

We obtained 400 panoramic radiographs (PRs) retrospectively from patients (242 females and 158 males; mean age 71.01 ± 7.96 years) who visited Seoul National University Dental Hospital from 2009 to 2022. PRs were collected from the digital panoramic machines of OP-100 (Instrumentarium Dental, Nahkelantie, Finland) with dimensions of 1976 × 976 pixels under condition of 70 kVp and 8–10 mA. In addition, PRs with dimensions of 2988 × 1468 pixels (resized as 1976 × 976 pixels) were obtained from Ray-alpha (Ray, Hwaseong-si, Korea) at 71 kVp and 12–14 mA. CT reference images were obtained using MDCT (Somatom Sensation 10, Siemens AG, Erlangen, Germany) under condition of 120 kVp and 130 mA. These images had voxel dimensions of 0.469 × 0.469 × 0.5 mm^3^, 512 × 512 pixels, and a 16-bit depth. In addition, another MDCT (Somatom Definition Edge, Siemens AG, Erlangen, Germany) was used to acquire images with voxel sizes of 0.37 × 0.37 × 0.6 mm^3^, dimensions of 512 × 512 pixels, and 12-bit depth at 120 kVp and 120 mA. This study was performed with approval from the institutional review board of Seoul National University Dental Hospital (ERI23031). The ethics committee approved a waiver of informed consent because this was a retrospective study. The study was performed in accordance with the Declaration of Helsinki.

**Figure 1:**
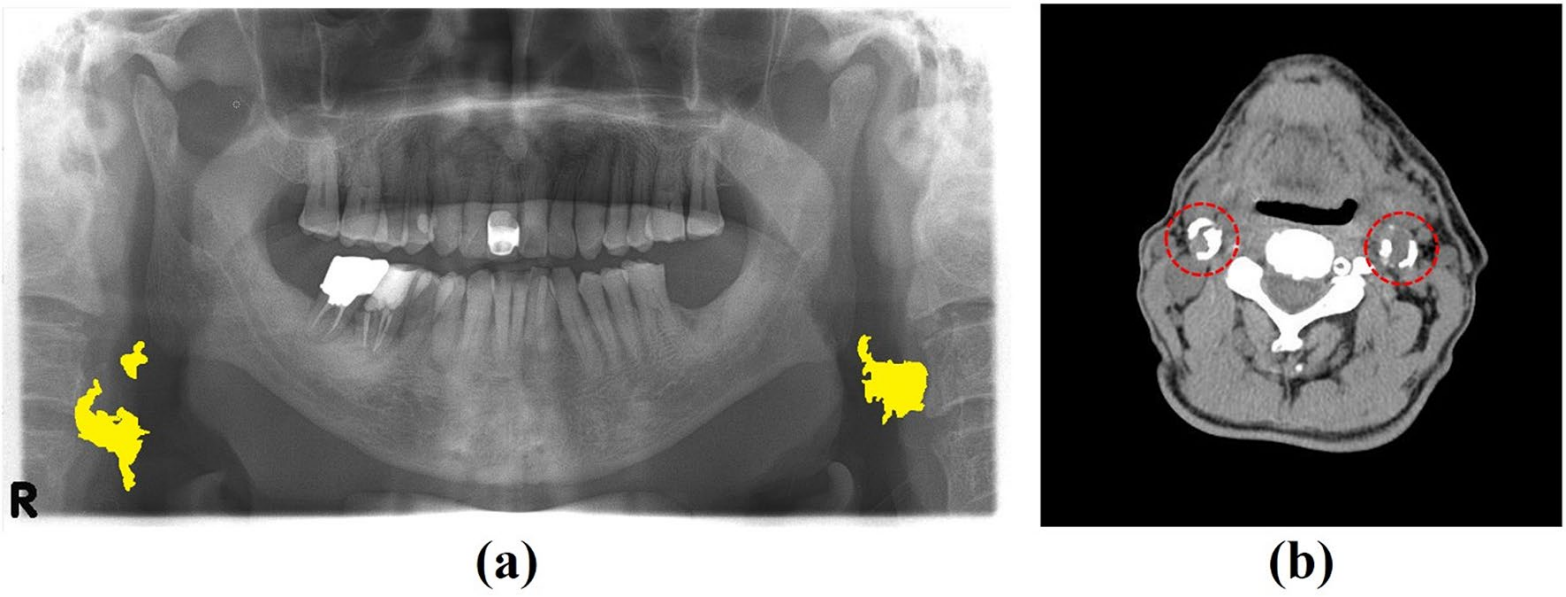
(**a**) Panoramic radiograph (PR) and (**b**) CT axial image for a patient. CT images were used as reference for CAC annotation on the PR. Yellow areas indicate carotid artery calcification lesions on the PR. Red dashed circles represent carotid artery calcification on the CT image.

A single PR for each patient was used for analysis. To accurately establish the ground truth for the presence and region of carotid artery calcification (CAC) on the PR, ground truth data was determined with reference to the CT image of the same patient, as CT is the standard imaging tool used to identify and quantify cardiovascular calcification^25^, and is superior to other imaging modalities for visualization of calcifications^26^ (Fig. 1). Inclusion criterion was the availability of a CT scan taken within one year before or after the PR. The PR was considered abnormal if calcification in the carotid artery was evident on both the PR and CT image, and as normal if no calcification in the carotid artery was observed on either the PR or CT image. Exclusion criteria were (a) inadequate image quality for diagnostic purposes on either the PR or CT image; (b) significant distortion or obscuration of the carotid artery region in the PR; and (c) extensive surgical procedures carried out near the area of interest. The data analysis of ROIs and CAC lesions is given in Supplementary Fig. S1.

**Figure 2:**
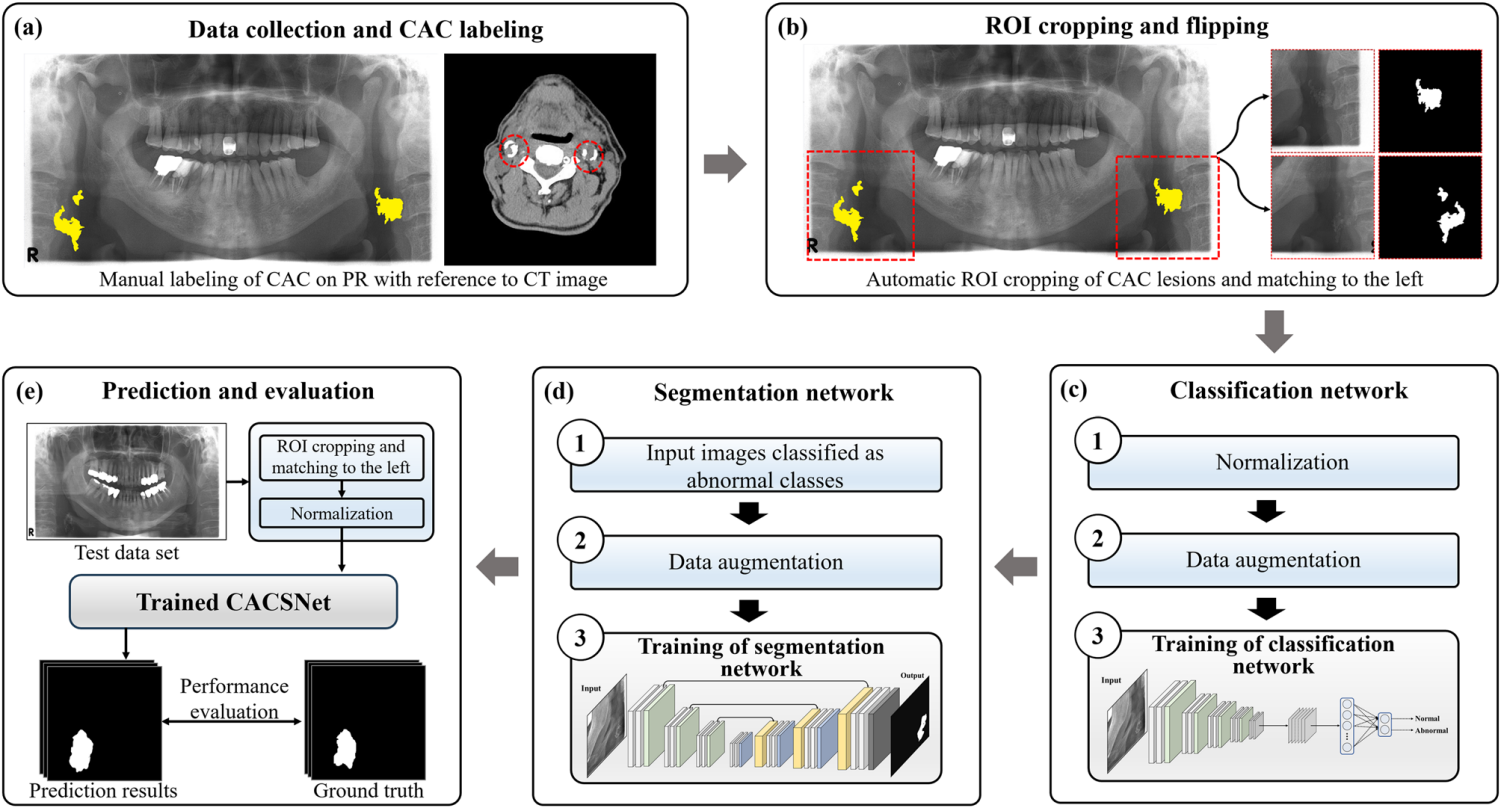
Overall procedures of the proposed method. (**a**) Data collection and carotid artery calcification (CAC) labeling with reference to CT image; (**b**) automatic ROI cropping and matching to the left; (**c**) training of the CAC classification network; (**d**) training of the CAC segmentation network; (**e**) prediction and evaluation processes of CACSNet.

Half of the 400 PRs were found to be abnormal cases with CAC at the left and/or right regions, while the other half were determined to be normal cases without CAC at the left or right regions (Table 1). An oral radiologist with more than ten years of experience manually annotated the CAC on PRs based on the CT reference image to ascertain the shape, location, and size of these for classification and segmentation using software (Supervisely OU, https://supervisely.com, Estonia) (Fig. 1). Two other oral radiologists with more than two decades of experience verified the annotated images with reference to CT images (Fig. 1). Left and right regions of interest (ROI) including CAC lesions were automatically cropped to 512 × 512 pixels at the bottom left and bottom right of the image, respectively. The right regions were horizontally flipped to match the left (Supplementary Table S1). The cropping size of the ROI was determined empirically to include the various CAC lesions. We applied Min–Max normalization techniques to the cropped images. The dataset was randomly divided into training, validation, and test sets at a 3:1:1 ratio (Table 1). Overall procedures are illustrated in Fig. 2.

**Table 1:** Dataset configuration of normal and abnormal panoramic radiographs used for deep learning.

Dataset	Normal patients (*N* = 200)		Abnormal patients (*N* = 200)	
	Left	Right	Left	Right
Training	120	120	75	73
Validation	40	40	25	25
Test	40	40	25	25
Total	200	200	125	123

### Network architecture of CACSNet

**Figure 3:**
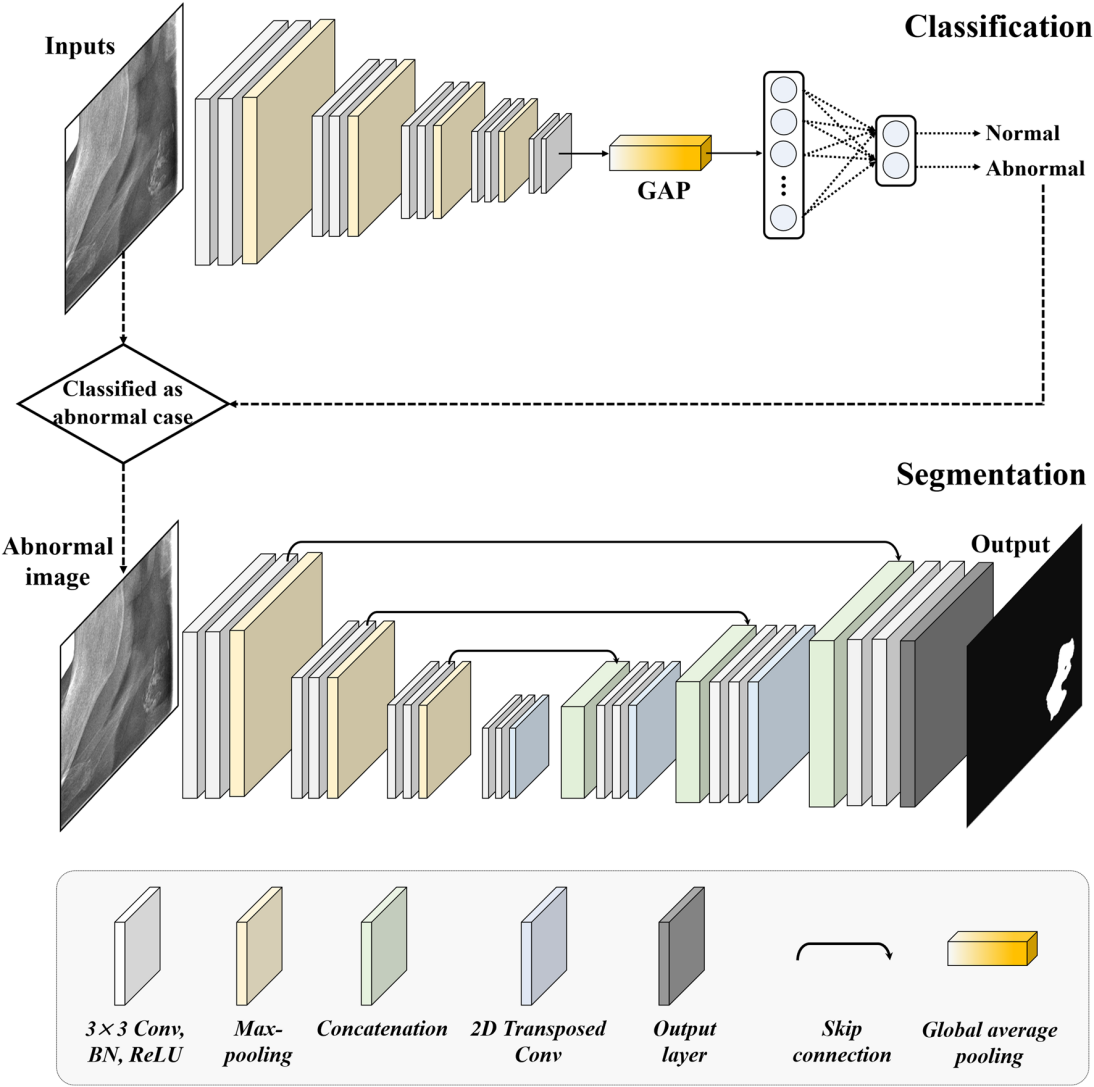
Overview of cascaded deep learning network (CACSNet) architecture. CACSNet consisted of CAC classification and segmentation networks to sequentially perform the classification and segmentation of CACs in PR.

We designed a cascaded deep learning network (CACSNet) that performs both classification and segmentation of CAC lesions (Fig. 2). The classification and segmentation networks for CACs are shown in Fig. 3. In the classification network of the CACSNet, PR images were classified as normal or abnormal images using the following CNN-based backbones: VGG16^27^, MobileNet v2^28^, ResNet101^29^, DenseNet121^30^, and EfficientNet-B4^31^. Global average pooling and sigmoid activation were used to output the classes. Only abnormal cases were used as input images to train the CAC segmentation network of CACSNet. The segmentation network was designed as an end-to-end encoder-decoder network with five CNN-based backbones to automatically segment CACs in PRs. The CNN-based backbones VGG16^27^, ResNet101^29^, DenseNet121^30^, SENet^32^, and EfficientNet-B4^31^ were used to extract informative features of CACs at the encoder in the segmentation network. At the decoder, a 3 × 3 convolution layer, batch normalization, rectified linear unit, and transposed convolution were used to recover the image resolution and refine the segmentation results. Furthermore, encoder features were transferred to corresponding decoder features to preserve fine-scale information by skip connections. To infer CAC regions at the output layer, a 3 × 3 convolution layer and sigmoid activation were used. The initial weights of all networks were adopted from ImageNet^33^ for transfer learning^34^.

We adopted binary cross-entropy loss (BCL) and Tversky loss (TVL)^35,36^ to train the classification and segmentation networks, respectively. BCL measures the difference in the average probability error between the ground truth and the prediction result. BCL is defined as

$$BCL\left(y,p\right) =-{\sum }_{i=1}^{N}{y}_{i}\text{log}\left({p}_{i}\right),$$

where *N,* $y$, and $p$ are the number of images, ground truth, and prediction results, respectively. TVL is widely used in segmentation tasks to measure the similarity between the ground truth and the prediction result^35,36^. TVL is different from Dice similarity coefficient loss (DSL) as it is designed to penalize false positives and false negatives differently, allowing control of the sensitivity of the loss function to false positives and false negatives. TVL is defined as

$$TVL\left(g,p\right)=1-\frac{\sum_{i}^{n}\left({g}_{i}\times {p}_{i}\right)}{\sum_{i}^{n}\left({g}_{i}\times {p}_{i}\right)+ \alpha \sum_{i}^{n}\left(\left(1-{g}_{i}\right)\times {p}_{i}\right)+\beta \sum_{i}^{n}\left({g}_{i}\times \left(1-{p}_{i}\right)\right)},$$

where $n$, $g$, and $p$ are the number of pixels, ground truth, and prediction result, respectively. $\alpha$ and $\beta$ are hyper-parameters to penalize false positives and false negatives, respectively.

The proposed networks were trained for 200 epochs with a mini-batch size of 4. Data augmentation was performed with rotation (− 30° to 30°), width and height shift (− 20 to 20%) of image size in horizontal and vertical axes, and zoom (0–20%). An Adam optimizer was used with ${\beta }_{0}=0.9$ and ${\beta }_{1}=0.999$. The learning rate was initially set as ${10}^{-3}$, and was reduced by half up to ${10}^{-6}$ when validation loss saturated for 25 epochs. Networks were implemented in Python3 using the Keras framework with TensorFlow backend, and trained on a workstation with an Intel i9-7900X CPU 3.3 GHz, 128 GB RAM, and two NVIDIA GeForce GTX 1080 Ti GPUs.

### Performance evaluation of CACSNet for classification and segmentation

We compared the CAC classification performances of CACSNet with different backbones (VGG16, MobileNet v2, ResNet101, DenseNet121, and EfficientNet-B4). To evaluate classification performance, we used the area under the receiver operating characteristic curve (AUC), accuracy ($\text{ACC}=\frac{TP+TN}{TP+TN+FP+FN}$), sensitivity ($\text{SEN}=\frac{TP}{TP+FN}$), and specificity ($\text{SPE}=\frac{TN}{TN+FP}$), where *TP*, *TN*, *FN*, and *FP* are abbreviations for true positives, true negatives, false negatives, and false positives, respectively. We also compared the segmentation performance of CACSNet with different backbones (VGG16, ResNet101, DenseNet121, SENet, and EfficientNet-B4). Jaccard index ($\text{JI}=\frac{TP}{TP+FP+FN}$), Dice similarity coefficient ($\text{DSC}=\frac{2\times TP}{2\times TP+FP+FN}$), precision ($\text{PR}=\frac{TP}{TP+FP}$), and recall ($\text{RC}=\frac{TP}{TP+FN}$) were used to evaluate the segmentation performance of CACSNet. Also, we calculated the intraclass correlation coefficient (ICC) of CAC areas between the ground truth and segmentation results of CACSNet with different backbones. A 95% confidence interval and statistical significance of AUC were analyzed using the DeLong’s test^37^, where the significant level was set at 0.05. Statistical analysis was conducted using RStudio (Version 2023.12.1 + 402; RStudio, PBC, Boston, MA, USA).

Deep learning models are often referred to as black boxes since users cannot comprehend the inner workings behind the model's prediction process^38^. Explainable AI has been developed to enhance our understanding of how AI systems make decisions and to increase the reliability of their predictions^39^. Gradient-weighted class activation mapping (Grad-CAM) is a technique that calculates node importance within convolutional layers using gradient information, and enhances the transparency of CNN-based models through visual explanations^40^. We used Grad-CAM to interpret the decision-making processes of the CAC classification network. Grad-CAM generated heatmaps of regions that deep learning models focused on when making a prediction^40^. Grad-CAM calculated gradients of the predicted class score on feature maps of the final convolutional layer and weighted summed them to provide a heatmap of the regions that contributed the most to the classification output^40^.

## Results

Classification and segmentation performances of the networks were evaluated using a test set not used for training. We tested several CNN-based backbones, namely VGG16, MobileNet v2, ResNet101, DenseNet121, and EfficientNet-B4 in the CAC classification network. We also evaluated several backbones as encoders in the CAC segmentation network, namely VGG16, ResNet101, DenseNet121, SENet, and EfficientNet-B4. For comparison experiments, all networks were trained with the same data augmentations under the same computational environments to guarantee a fair comparison.

**Figure 4:**
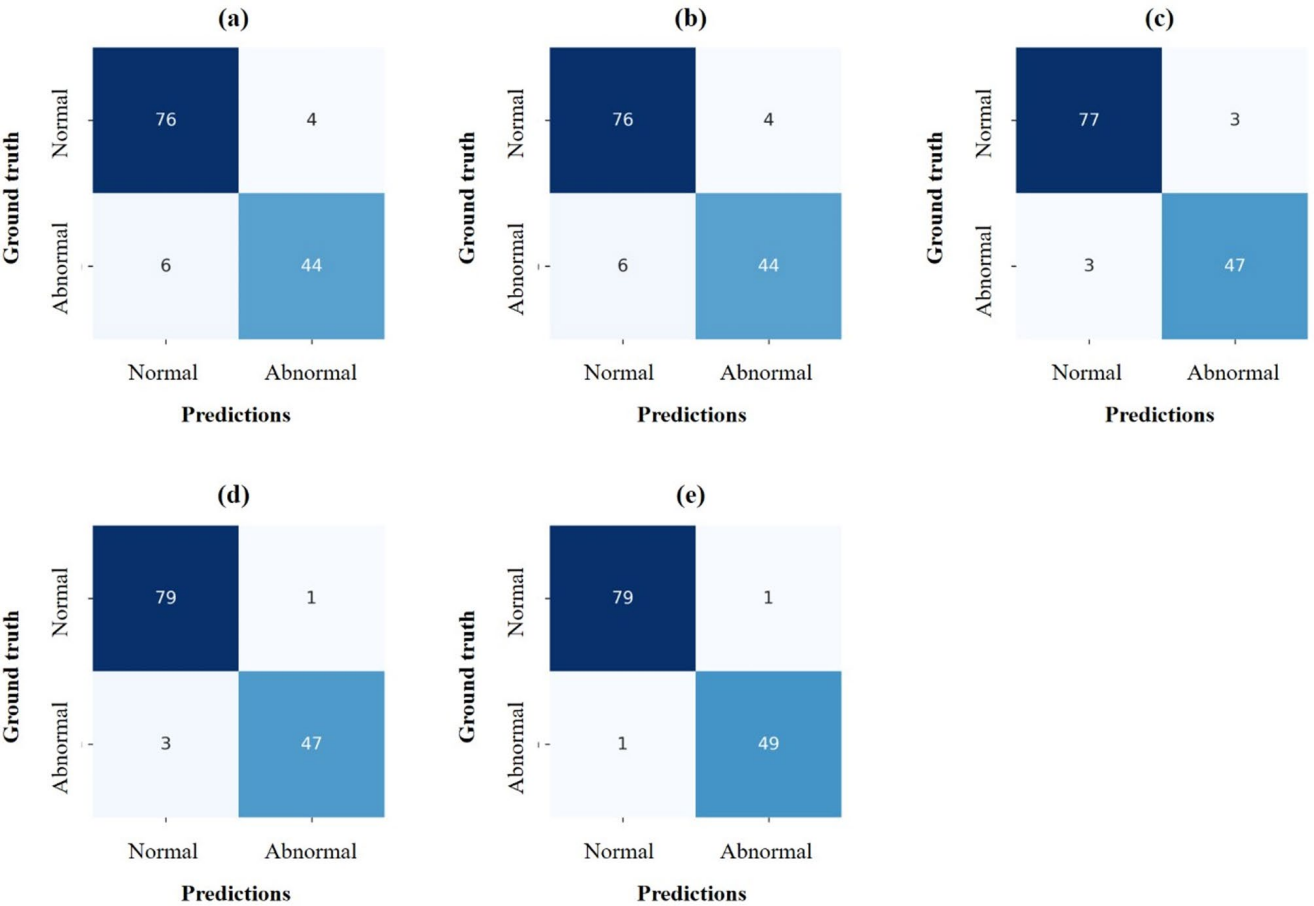
Confusion matrices for classification of carotid artery calcification by CACSNet with backbones of (**a**) VGG16, (**b**) MobileNet v2, (**c**) ResNet101, (**d**) DenseNet121, and (**e**) EfficientNet-B4. The false positives and negatives by EfficientNet-B4 are shown in the Supplementary Fig. S2.

**Figure 5:**
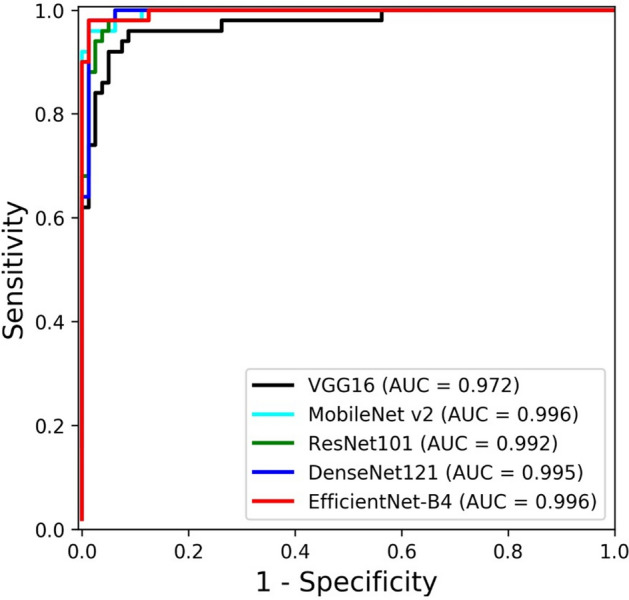
Receiver operating characteristic (ROC) curves for classification of carotid artery calcification by CACSNet with backbones of VGG16, MobileNet v2, ResNet101, DenseNet121, and EfficientNet-B4. There was a significant difference in AUC between VGG16 and MobileNet v2 (*p* < 0.05) and between VGG16 and EfficientNet-B4 (*p* < 0.05).

**Figure 6:**
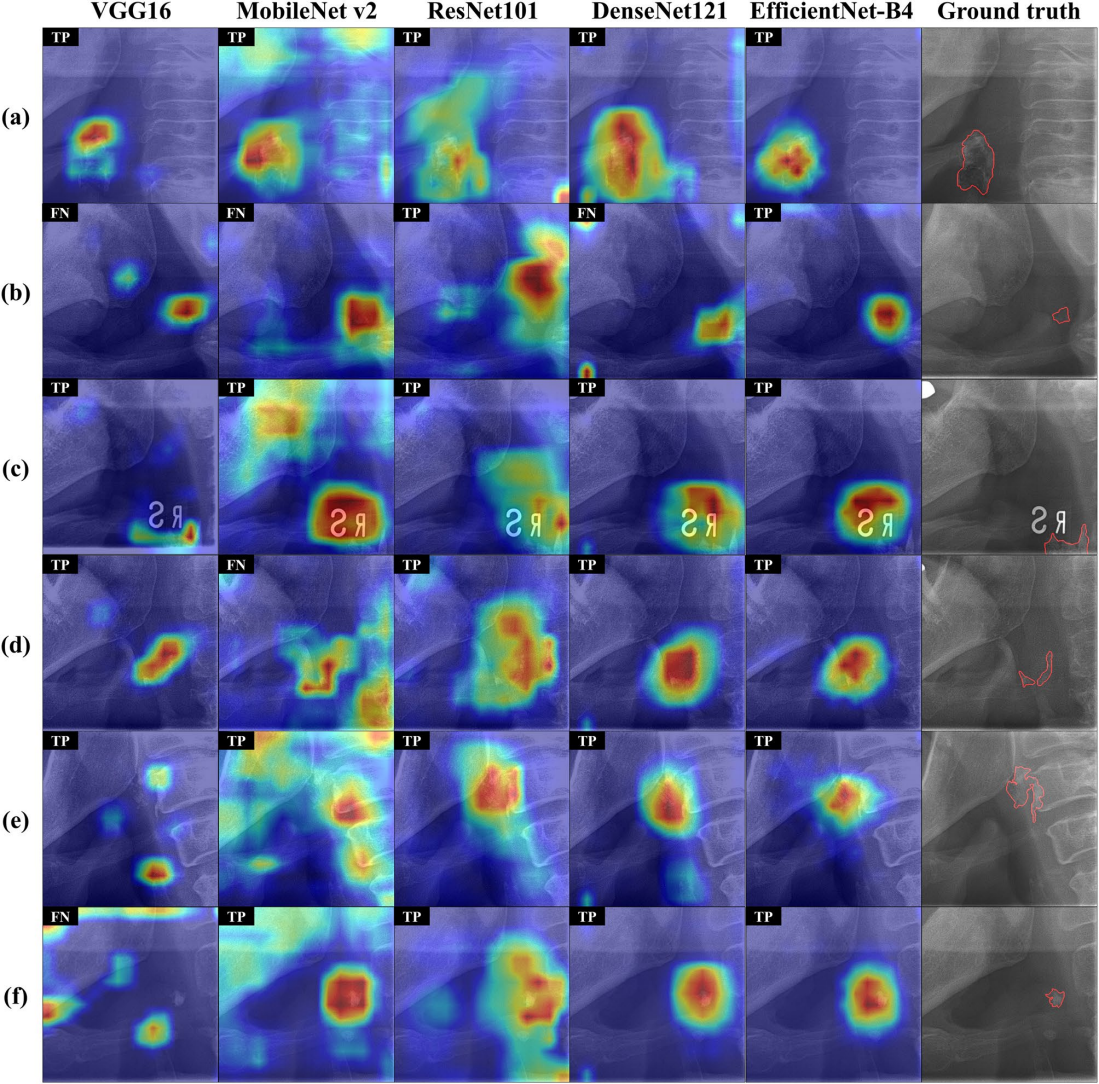
Grad-CAM results of classification by CACSNet with backbones of VGG16, MobileNet v2, ResNet101, DenseNet121, and EfficientNet-B4. Bright red indicates that the corresponding region contributes strongly to the decision of the model. Grad-CAM results for (**a**–**c**) CACs with large variations in size, shape, and location, and (**d**–**f**) those overlapping with surrounding anatomical structures. TP and FN indicate true positives and false negatives, respectively, after classification. The ground truth represents the original image with annotation (red line) for each case.

Table 2 shows the quantitative results of CAC classification performance according to backbones. CACSNet with EfficientNet-B4 had fewer false positives and negatives than CACSNet with the other backbones (Fig. 4), and achieved the highest accuracy, sensitivity, and specificity values of 0.985, 0.980, and 0.988, respectively (Table 2). CACSNet with EfficientNet-B4 and MobileNet v2 obtained the highest AUC value of 0.996 for classification (Fig. 5). There was a significant difference in AUC between VGG16 and MobileNet v2 (*p* < 0.05) and between VGG16 and EfficientNet-B4 (*p* < 0.05). To interpret the decision-making processes of CACSNet, Grad-CAM was used to visualize activation regions that contributed the most to the classification output. CACSNet with EfficientNet-B4 focused more densely on CAC lesions than CACSNet with the other backbones (Fig. 6). Heatmaps for EfficientNet-B4 showed more sensitive and robust activation on CACs with large variations in shape (Fig. 6a), size (Fig. 6b), and locations (Fig. 6c), whereas those with the other backbones showed sparse or wide activations in irrelevant anatomical structures such as near the hyoid bone, cervical spine, and mandible. Heatmaps of EfficientNet-B4 also focused more densely on specific regions of CACs that overlapped with surrounding anatomical structures than the other backbones (Fig. 6d–f). In Fig. 6, sparse and wider activation regions in the heatmaps, predicted as false negatives by the other backbones, were observed at irrelevant anatomical structures. As a result, CACSNet with EfficientNet-B4 provided classification outputs with a more detailed, precise, and accurate focus on CACs with large variations in size, shape, and location, and those overlapped with surrounding anatomical structures than the other networks.

**Table 2:** Classification performance of CACSNet with different backbones for carotid artery calcification.

Backbone	AUC	ACC	SEN	SPE
VGG16	0.972*^,†^ [0.945, 1.000]	0.923	0.880	0.950
MobileNet v2	0.996 [0.990, 1.000]	0.962	0.920	0.988
ResNet101	0.992 [0.982, 1.000]	0.954	0.940	0.963
DenseNet121	0.995 [0.985, 1.000]	0.969	0.940	0.988
EfficientNet-B4	0.996 [0.991, 1.000]	0.985	0.980	0.988

The square brackets indicate the 95% confidence interval of AUC. Significant differences in AUC *between VGG16 and MobileNet v2 (*p* < 0.05), and ^†^between VGG16 and EfficientNet-B4 (*p* < 0.05).

To determine optimal $\alpha$ and $\beta$ weights of Tversky loss in the segmentation network of CACSNet, an ablation study was performed by asymmetrically changing values between the two weights. Table 3 shows the CAC segmentation performance when adjusting $\alpha$ and $\beta$ weights of Tversky loss in CACSNet with EfficientNet-B4. As the $\alpha$ weight gradually increased, the precision value increased, but the recall value decreased. As the $\beta$ weight gradually increased, the recall value increased, but the precision value decreased. These results indicate a trade-off between precision and recall according to the $\alpha$ and $\beta$ weight values of Tversky loss. With weights of $\alpha =0.6$ and $\beta =0.4$, CACSNet with EfficientNet-B4 achieved the highest JI and DSC values of 0.595 and 0.722, respectively, while maintaining a balance between precision and recall. An additional ablation study was performed by changing loss functions including BCL, Jaccard index loss (JIL), DSL, and TVL in the segmentation network (Table 4). TVL achieved the highest JI and DSC values compared with the other loss functions in CACSNet with EfficientNet-B4.

**Table 3:** Ablation study of $\alpha$ and $\beta$ weights of Tversky loss of CACSNet with EfficientNet-B4.

Weights	JI	DSC	Precision	Recall	ICC
*α* = 0.1, *β* = 0.9	0.497 ± 0.174	0.646 ± 0.160	0.577 ± 0.193	0.863 ± 0.215	0.764
*α* = 0.2, *β* = 0.8	0.559 ± 0.186	0.697 ± 0.171	0.644 ± 0.184	0.838 ± 0.214	0.792
*α* = 0.3, *β* = 0.7	0.577 ± 0.207	0.708 ± 0.185	0.708 ± 0.183	0.787 ± 0.228	0.747
*α* = 0.4, *β* = 0.6	0.581 ± 0.216	0.709 ± 0.200	0.710 ± 0.207	0.774 ± 0.239	0.767
*α* = 0.5, *β* = 0.5	0.568 ± 0.192	0.704 ± 0.170	0.707 ± 0.197	0.791 ± 0.212	0.722
*α* = 0.6, *β* = 0.4	0.595 ± 0.206	0.722 ± 0.192	0.749 ± 0.204	0.756 ± 0.224	0.772
*α* = 0.7, *β* = 0.3	0.538 ± 0.231	0.665 ± 0.232	0.779 ± 0.237	0.629 ± 0.248	0.676
*α* = 0.8, *β* = 0.2	0.559 ± 0.226	0.684 ± 0.232	0.794 ± 0.222	0.652 ± 0.245	0.674
*α* = 0.9, *β* = 0.1	0.518 ± 0.213	0.651 ± 0.230	0.824 ± 0.259	0.561 ± 0.227	0.617

**Table 4:** Ablation study for loss functions of CACSNet with EfficientNet-B4.

Loss functions	JI	DSC	Precision	Recall	ICC
BCL	0.562 ± 0.227	0.688 ± 0.222	0.799 ± 0.172	0.688 ± 0.255	0.661
JIL	0.559 ± 0.186	0.697 ± 0.172	0.644 ± 0.214	0.838 ± 0.214	0.792
DSL	0.568 ± 0.192	0.704 ± 0.170	0.707 ± 0.197	0.791 ± 0.212	0.722
TVL	0.595 ± 0.206	0.722 ± 0.192	0.749 ± 0.204	0.756 ± 0.224	0.772

*BCL* binary cross-entropy loss, *JIL* Jaccard index loss, *DSL* Dice similarity coefficient loss, *TVL* Tversky loss.

**Figure 7:**
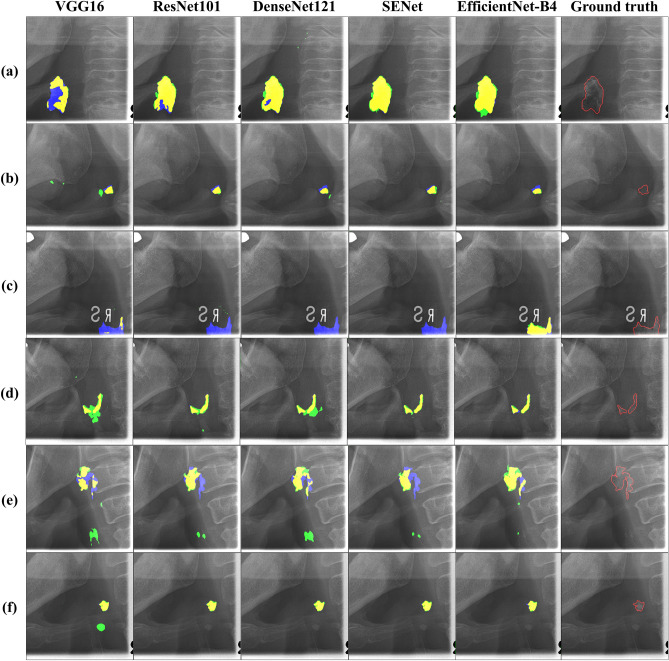
Segmentation results by CACSNet with backbones of VGG16, ResNet101, DenseNet121, SENet, and EfficientNet-B4. Blue, green, and yellow regions indicate false negatives, false positives, and true positives, respectively. Segmentation results for (**a**–**c**) CACs with large variations in size, shape, and location, and (**d**–**f**) those overlapping with surrounding anatomical structures. The ground truth represents the original image with annotation (red line) for each case.

**Figure 8:**
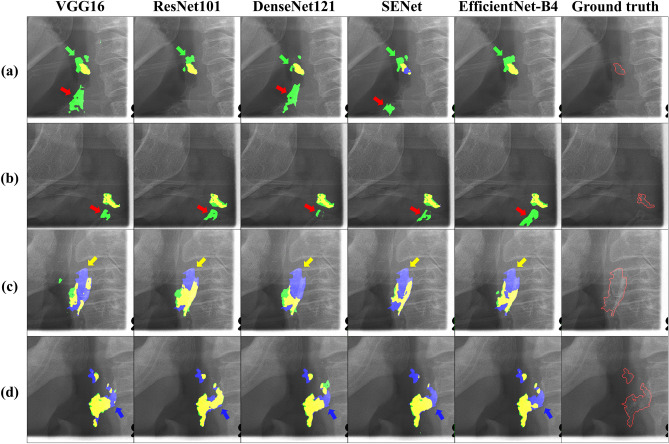
Segmentation errors predicted by CACSNet with different backbones. Blue, green, and yellow regions indicate false negatives, false positives, and true positives, respectively. (**a**) CACSNet predicted the hyoid bone (green arrow) and the thyroid cartilage (red arrow) as CAC (false positives), (**b**) CACSNet predicted the thyroid cartilage (red arrow) as CAC (false positives), (**c**) CACSNet could not accurately predict CAC regions (yellow arrow) overlapping with the cervical spine (false negatives), (**d**) CACSNet could not accurately predict CAC regions (blue arrow) overlapping with the posterior pharyngeal wall (false negatives). The ground truth represents the original image with annotation (red line) for each case.

Table 5 shows the CAC segmentation performance of CACSNet with different backbones. CACSNet with EfficientNet-B4 demonstrated the best segmentation performance (JI, DSC, precision, and recall of 0.595, 0.722, 0.749, and 0.756, respectively). Compared to CACSNet with the other backbones, CACSNet with EfficientNet-B4 showed better segmentation results with fewer false positives and negatives for CAC lesions with large variations in size, shape, and location (Fig. 7a–c), and those that overlapped with surrounding anatomical structures (Fig. 7d–f). As a result, CACSNet with EfficientNet-B4 more accurately predicted CAC lesions with large morphological variations and overlaps. Nonetheless, CACSNet showed false positive errors for CACs near the hyoid bone (Fig. 8a) and the thyroid cartilage (Fig. 8b), and false negatives for CACs that overlapped with the cervical spine (Fig. 8c) and the posterior pharyngeal wall (Fig. 8d). We showed the segmentation performance of CACSNet with EfficientNet-B4 according to the size and mean pixel values of CAC lesions in Supplementary Fig. S3.

**Table 5:** Segmentation performance of CACSNet with different backbones for carotid artery calcification.

Backbone	JI	DSC	Precision	Recall	ICC
VGG16	0.514 ± 0.235	0.643 ± 0.238	0.674 ± 0.225	0.708 ± 0.281	0.569
ResNet101	0.558 ± 0.217	0.687 ± 0.217	0.743 ± 0.200	0.719 ± 0.263	0.772
DenseNet121	0.538 ± 0.243	0.662 ± 0.247	0.697 ± 0.226	0.719 ± 0.284	0.678
SENet	0.566 ± 0.241	0.688 ± 0.235	0.706 ± 0.233	0.726 ± 0.272	0.660
EfficientNet-B4	0.595 ± 0.206	0.722 ± 0.192	0.749 ± 0.204	0.756 ± 0.224	0.772

**Figure 9:**
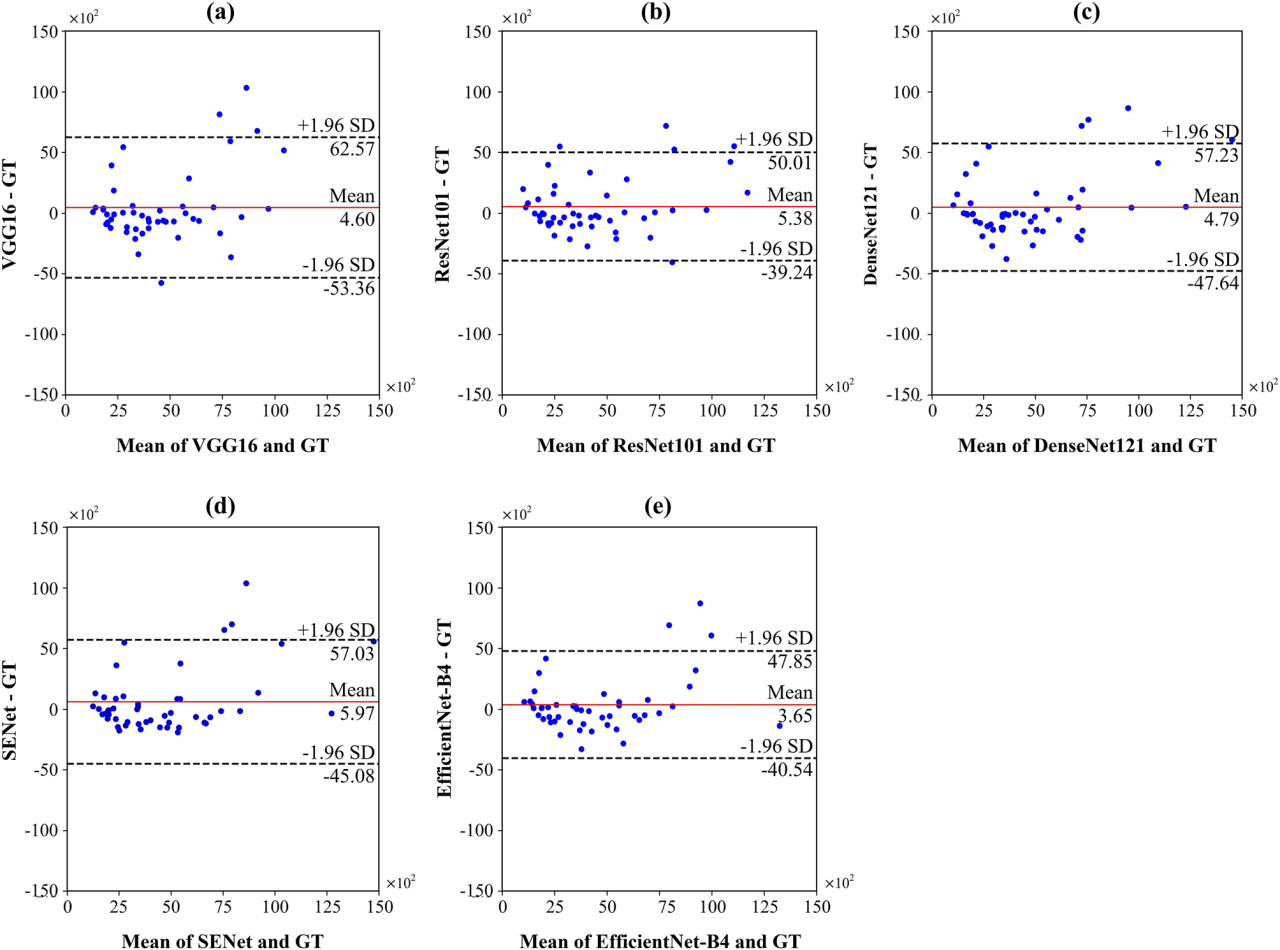
Bland–Altman plots of the ground truth and segmentation results by CACSNet with backbones of (**a**) VGG16, (**b**) ResNet101, (**c**) DenseNet121, (**d**) SENet, and (**e**) EfficientNet-B4. Blue dots are area differences (pixels) between the ground truth and segmentation results. Black dashed and red lines are 95% limits of agreement and mean difference, respectively.

**Figure 10:**
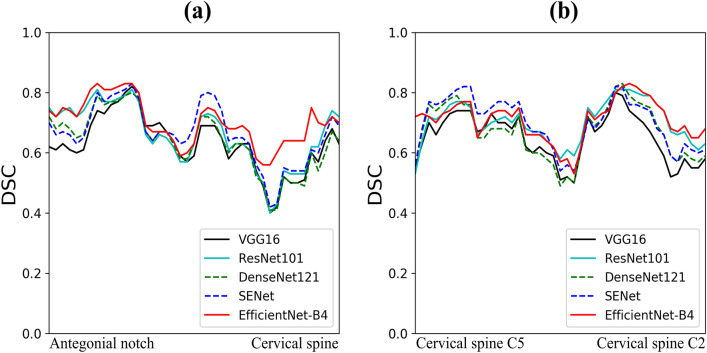
Line plots for Dice similarity coefficient score (DSC) of segmentation results by CACSNet with backbones of VGG16 (black), ResNet101 (cyan), DenseNet121 (green), SENet (blue), and EfficientNet-B4 (red) according to (**a**) CAC horizontal locations (from the antegonial notch of the mandible to the cervical spine); DSC values are plotted according to *x* coordinates of the center of mass for the ground truth image, (**b**) CAC vertical locations (from the cervical spine C5 to C2); DSC values are plotted according to *y* coordinates of the center of mass for the ground truth image.

Bland–Altman plots of area differences between the ground truth and segmentation predictions from CACSNets with different backbones revealed that EfficientNet-B4 showed higher linear relationships and better agreement limits than the other backbones and presented consistent segmentation performance across various sizes of CACs (Fig. 9). EfficientNet-B4 also showed smaller variation in segmentation performance than the other backbones when DSC values were plotted according to horizontal or vertical locations of CACs (Fig. 10). Furthermore, the classification network of CACSNet improved segmentation performance by reducing false negative CAC segmentation results (Table 6). Therefore, CACSNet with EfficientNet-B4 was robust to large morphological variations and overlap of CAC lesions with anatomical structures over the entire posterior inferior region of the mandibular angle.

**Table 6:** Segmentation performance of CACSNet with EfficientNet-B4 with and without a classification network.

	JI	DSC	Precision	Recall	ICC
Without classification	0.543 ± 0.262	0.658 ± 0.275	0.752 ± 0.215	0.685 ± 0.318	0.823
With classification	0.595 ± 0.206	0.722 ± 0.192	0.749 ± 0.204	0.756 ± 0.224	0.772

## Discussion

Stroke is the second-leading cause of death worldwide^1^. A significant percentage of stroke is caused by carotid artery atherosclerosis; early detection of carotid artery atherosclerosis can, therefore, reduce the risk of stroke^3,41^. PR is a routinely used imaging technique in dental practice that can be used to visualize calcified atheromas of the carotid artery^15^. Therefore, PR could potentially be used to screen for and detect non-symptomatic CAC patients among the general population^42^. However, the diagnostic capacity of conventional PRs is not sufficient for reliable and precise evaluation of CAC^17,43^. In addition, dentists may not pay close attention to structures outside the oral cavity such as the carotid arteries. As a result, PR is not commonly utilized for the purpose of screening for CAC in the oral healthcare setting. If CAC can be accurately diagnosed on PR with the help of an automatic method of detection (or classification) and segmentation, dental professionals can screen patients at risk of stroke more easily, and ensure that patients receive appropriate treatment^44^. Therefore, we developed CACSNet for automatic robust classification and segmentation of CACs with large variations in size, shape, and location, as well as CACs overlapping with surrounding anatomical structures.

Unlike images commonly used for computer vision tasks, PRs are consistently captured with patients positioned at a specific location and angle, and consequently, structures on PRs tend to exhibit consistent patterns in similar locations^45^. Despite this, CAC is characterized as an irregular nodular radiopacity adjacent to the cervical vertebrae, close to the intervertebral space of C3 and C4, and positioned posteroinferiorly to the mandibular angle and the hyoid bone in the context of PR^46^, however, their locations are variable and can be outside of the region covered by the PR^47^. Distinguishing CAC with certainty is a difficult task and providing accurate ground truth labeling of lesions in 2D projection images (i.e., PRs) makes applications of deep learning for segmentation challenging because it is difficult to accurately discriminate lesions from other surrounding anatomical structures. Furthermore, CACs vary widely in their size, shape, and location within the carotid arteries, as well as stage and type of calcification. CACs appear in various and undefined shapes, such as irregular, heterogeneous, verticolinear radiopacity in PR^16^, and are mostly circular when small and linear or thin rectangular when enlarged^48^. As plaque size increases, the calcification area also expands^49^, typically ranging from 1.5 to 4.0 cm^50^. Sometimes, the CAC is very small and cannot be identified on a PR^43^. Although differential diagnosis is conducted based on the locations and morphologies of structures, the ability to diagnose CACs by conventional PRs is limited.

Differential diagnosis of CAC involves distinguishing both anatomical and pathological radiopacities. Anatomical structures include the hyoid bone, styloid process, stylohyoid ligament, stylomandibular ligament, thyroid cartilage, triticeous cartilage, epiglottis, and anterior tubercle of the atlas vertebrae^16^. Pathological structures encompass lymph nodes, phleboliths, submandibular salivary glands sialoliths, loose body, and tonsilloliths^16^. Discriminating the CAC from these structures on PRs remains challenging as overlap with other structures leads to increased uncertainty when labeling the image. To improve the performance of deep learning models, it is imperative that the ground truth data used for model training be highly accurate. Previous studies have attempted to overcome these challenges through collaborative efforts among multiple researchers^20,21^. In this study, we improved labeling accuracy by using only abnormal images that were unequivocally identified as containing CAC in CT images. CT is recognized as the most advanced non-invasive tool for detecting vascular calcification and is a clinically accessible imaging technique^51^. It is considered the standard imaging tool for the identification and quantification of cardiovascular calcification^25^, and surpasses other imaging modalities for visualizing calcification^26^. Providing accurate ground truth data is essential for guaranteeing the high performance of deep learning models.

Before the widespread application of deep learning, traditional approaches were used for the automatic detection of CAC on PRs. Fuzzy image contrast enhancement and an algebraic image operator were used to extract a calcification region brighter than its neighbor; the detection rate of this method was 50%^52^. One study incorporated a support vector machine to reduce misdetection, and the number of false positive cases decreased to a 75% level of the previous method^53^. A method for detecting CACs using a top-hat filter showed a sensitivity of 93.6% with 4.4 false positives per image by reducing the number of false positives with a rule-based approach and support vector machine^54^. Some researchers have attempted to automate the detection of CAC in PRs based on deep learning networks^20,21^. Deep learning models achieved an AUC of 0.83, accuracy of 0.83, sensitivity of 0.75, specificity of 0.80^20^ or accuracy of 0.94, sensitivity of 0.82, and specificity of 0.97^21^. Our deep learning model with the EfficientNet-B4 backbone achieved an AUC of 0.996, accuracy of 0.985, sensitivity of 0.980, and specificity of 0.988 for CAC classification, demonstrating superior performance to previous models.

In the context of CAC segmentation on PRs, our research is pioneering, and direct benchmarks for comparison are therefore lacking. CACSNet with EfficientNet-B4 backbone achieved a JI of 0.595, DSC of 0.722, precision of 0.749, and recall of 0.756. Several investigations of CAC segmentation in images other than PRs using deep learning models have been conducted^22–24,55–57^. The deep learning models achieved a DSC of 0.795 in CT angiographic images^23^, a DSC of 0.9381 in ultrasound images^24^, and a DSC of 0.78, precision of 0.76, and recall of 0.8 in MR images^22^. Therefore, compared with previous studies performed on other modalities, CACSNet demonstrated comparable segmentation performance for CACs on PRs.

In this study, we applied a deep learning model using U-Net architecture in CACSNet for the segmentation of CAC in PRs. U-Net architecture includes a contracting path that captures contextual information and a symmetric expanding path that allows for precise localization of segmented areas^58^. We tested five validated networks (VGG16^27^, ResNet101^29^, DenseNet121^30^, SENet^32^, and EfficientNet-B4^31^) as backbones of CACSNet; EfficientNet-B4^31^ showed the best performance. EfficientNet has previously been reported to demonstrate exceptional performance in image classification tasks, surpassing previous state-of-the-art models using benchmarks such as ImageNet^33^.

In deep learning-based image segmentation, data imbalance is one of the primary challenges^59^. Especially in medical imaging, lesions often occupy a very small fraction of the entire image, leading to significant data imbalance^60^. This is reflected in high precision and low recall values^60^. To address this, we incorporated a Tversky loss function in our network. By adjusting the hyper-parameters $\alpha$ and $\beta$ of Tversky loss, false positives and false negatives can be balanced^35^. Because $\alpha$ penalizes false positives, increasing this value trains the network to reduce false positives and leads to higher precision. In our dataset, the balance point between precision and recall was achieved when $\alpha$ and $\beta$ were set to 0.6 and 0.4, respectively. Furthermore, the classification network of the cascaded deep learning networks improved segmentation performance by reducing false negative CAC segmentation results. Despite using significantly fewer data than typical deep learning research, the performance of our model was impressive. We attribute this in part to the benefits of transfer learning^34^ and data augmentation^61^, which we utilized to overcome the limitation of a small dataset. As a result, CACSNet demonstrated the robustness of segmentation to morphological variation and overlap of CAC lesions over the entire posterior inferior region of the mandibular angle in PRs.

To date, no automatic segmentation studies of CAC lesions in PRs using deep learning have been published, but there are many studies of segmentation of CACs on images taken using other imaging modalities^22–24,55–57^. This is likely due to the limited diagnostic accuracy of conventional PRs in assessing CAC, coupled with the difficulty in accurately labeling ground truth for CAC lesions on PRs. Consequently, our approach in this study involved refining labeling precision by exclusively incorporating images in which CAC was clearly identified with reference to CT images.

Segmentation of the CACs in PRs has proven beneficial for quantitatively assessing cardiovascular disease risk by providing detailed information about the size, location, and shape of the CACs. Quantifying the areas of carotid plaques in ultrasound images is commonly utilized to evaluate the risk of cardiovascular disease^62^. Measurements of the CAC lesions in PRs were correlated with the degree of carotid artery stenosis and the resistive index calculated from Doppler ultrasonography^63^. The volume of calcium in carotid plaques represents an independent marker for luminal stenosis and ischemic symptoms in CT images^10^. The size information of the CACs, such as volumes or areas, is critical for assessing cerebrovascular risk, planning therapeutic interventions^64^, and evaluating treatment outcomes^65^. The location information of the CACs can be used to assess the risk of cerebrovascular disease^66^ and predict the progression rate of carotid plaque^67^. The incidence of cerebrovascular events is assessed by analyzing the morphological characterization of the CACs^66^. Monitoring the longitudinal changes in the CACs allows the assessment of vascular disease risk and the efficacy of interventions^68^. Therefore, the segmentation of the CACs in PRs can be used to aid in diagnosing, assessing, and managing cerebrovascular risks.

Our study had several limitations. First, the size of the dataset used for deep learning was relatively small. The proposed method needs to be evaluated on PR datasets from more patients with various CACs with large variations in size, shape, and location, as well as CAC overlapping with anatomical structures. The CACSNet sometimes incorrectly predicts CACs near the hyoid bone, thyroid cartilage, and triticeous cartilage (false positives), which are the anatomical structures most frequently confused with CACs on PRs^69^, and those overlapping with the cervical spine and posterior pharyngeal (false negatives). Second, CACSNet may not be widely generalizable due to the use of PRs acquired from a single institution. It is necessary to improve generalizability using large PR datasets acquired under various imaging conditions from multi-centers using various devices. Last, we designed a cascaded deep learning model consisting of classification and segmentation networks; this model requires more computational time than single-stage deep learning models. In future studies, we intend to combine classification and segmentation networks in a single-stage deep learning model based on multi-task learning.

## Conclusions

We developed a cascaded deep learning network (CACSNet) to automatically and robustly segment CAC in PRs; ground truth labeling was accurately determined with reference to CT images. CACSNet demonstrated the robustness of classification and segmentation of CAC lesions to large morphological variations and overlaps with surrounding structures over the entire posterior inferior region of the mandibular angle in PRs. Accurate diagnosis of CAC in PRs using an automatic method with high precision can help dental professionals to screen patients at risk of stroke more easily. Future studies should focus on improving the segmentation performance of our method for CAC in PRs by employing advanced deep learning models and data augmentation techniques based on generative AI, along with incorporating more extensive datasets from multiple centers and devices.

### Supplementary Information

Supplementary Information.

## Data Availability

The datasets generated and/or analyzed during the current study are not publicly available due to restrictions of the Institutional Review Board (IRB) of Seoul National University Dental Hospital to protect patients’ privacy, but are available from the corresponding author on reasonable request.
