# Supplementary material for: CACSNet for automatic robust classification and segmentation of carotid artery calcification on panoramic radiographs using a cascaded deep learning network
Source: Sci Rep. 2024 Jun 17;14:13894. doi: 10.1038/s41598-024-64265-4 (PMC11183138; doi:10.1038/s41598-024-64265-4)
Supplement: Supplementary file 1 — Supplementary Information. [file 41598_2024_64265_MOESM1_ESM.pdf]

Supplementary Materials

A. Data analysis of ROIs and CAC lesions

The sizes of the CAC lesions are calculated by the number of pixels of the CAC lesions (Fig. S1a). We observe that the CAC lesions have a wide range of sizes in pixels in Fig. S1a. The large variations in size, as well as variations in shape and location and their overlapping with surrounding anatomical structures, made it difficult to identify CACs accurately.

Fig. S1b shows a density plot of pixel values (gray levels) in the CAC lesions and the ROIs. The pixel values in the ROIs (blue area) and the pixel values in the CAC lesions (orange area) are densely distributed from 50 to 150 and 50 to 100, respectively. The high-density areas of CAC lesions in the plot overlapped with those of the ROIs, which made it difficult to identify CACs in ROIs due to their low contrast between CACs and surrounding anatomical structures.

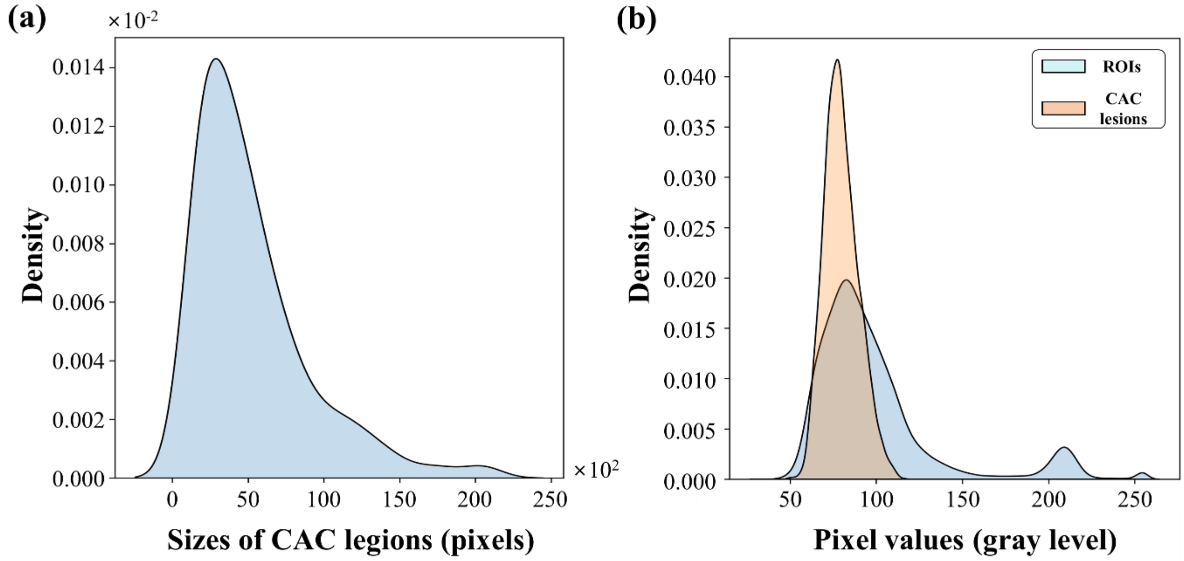

Figure S1. The density plots (a) for sizes of the CAC lesions, and (b) for pixel values of the CAC lesions and the ROIs.

B. The false negative case by EfficientNet-B4

Despite the presence of the CAC in the ROI, EfficientNet-B4 classified it as not having the CAC in the false negative case. The Grad-CAM of EfficientNet-B4 showed sparse or widespread activations in irrelevant anatomical structures in this case (Fig. S2a). The CACs in the false negative case showed a thin and horizontally elongated shape, and the EfficientNet-B4 might misidentify the CACs as a part of the vertebrae structure (Fig. S2a).

C. The false positive case by EfficientNet-B4

There were no CACs in the ROI, but EfficientNet-B4 classified it as having the CAC in the false positive case. The Grad-CAM of EfficientNet-B4 activated the greater horn of the hyoid bone falsely (Fig. S2b)

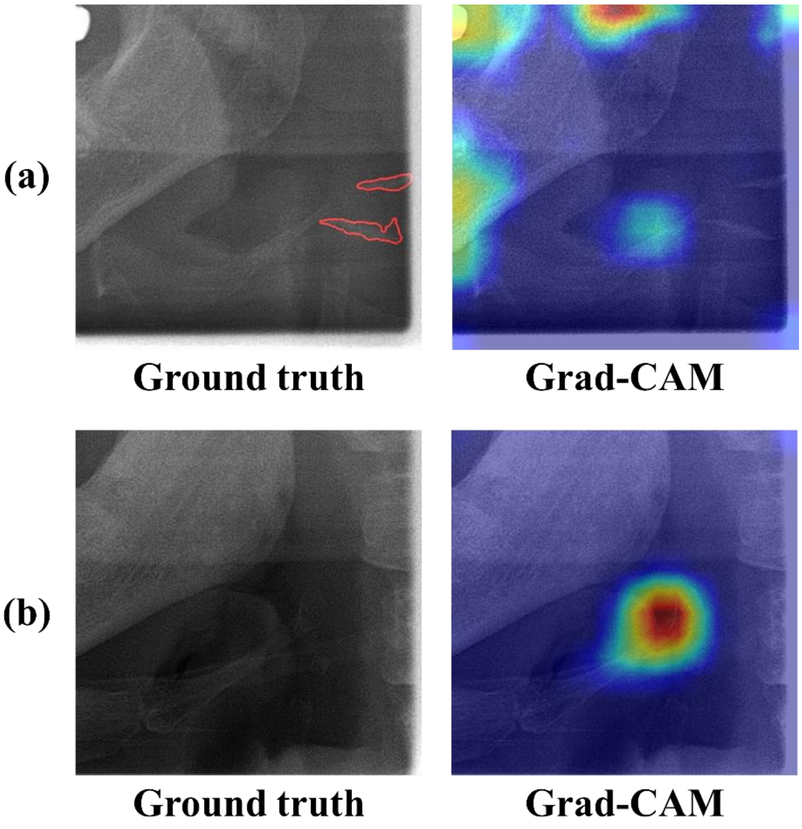

Figure S2. (a) False negative and (b) false positive cases predicted by EfficientNet-B4.

D. Ablation study for the effectiveness of horizontal flipping to match the left

We observe that CACSNet with horizontal flipping achieves higher performances than that without horizontal flipping (Table S1).

Table S1. Ablation study for the effectiveness of the horizontal flipping to match the left in CACSNet.

Horizontal flipping	JI	DSC	Precision	Recall	ICC
Without	0.568 ± 0.222	0.693 ± 0.237	0.721 ± 0.237	0.722 ± 0.250	0.780
With	0.595 ± 0.206	0.722 ± 0.192	0.749 ± 0.204	0.756 ± 0.224	0.772

E. Segmentation performance of CACSNet with EfficientNet-B4 according to the sizes and mean pixel values of CAC lesions

We evaluated the performance of CACSNet with EfficientNet-B4 according to the sizes and mean pixel values of CAC lesions (Fig. S3). The CACSNet with EfficientNet-B4 achieved consistent segmentation performance over various sizes and mean pixel values of the CAC lesions.

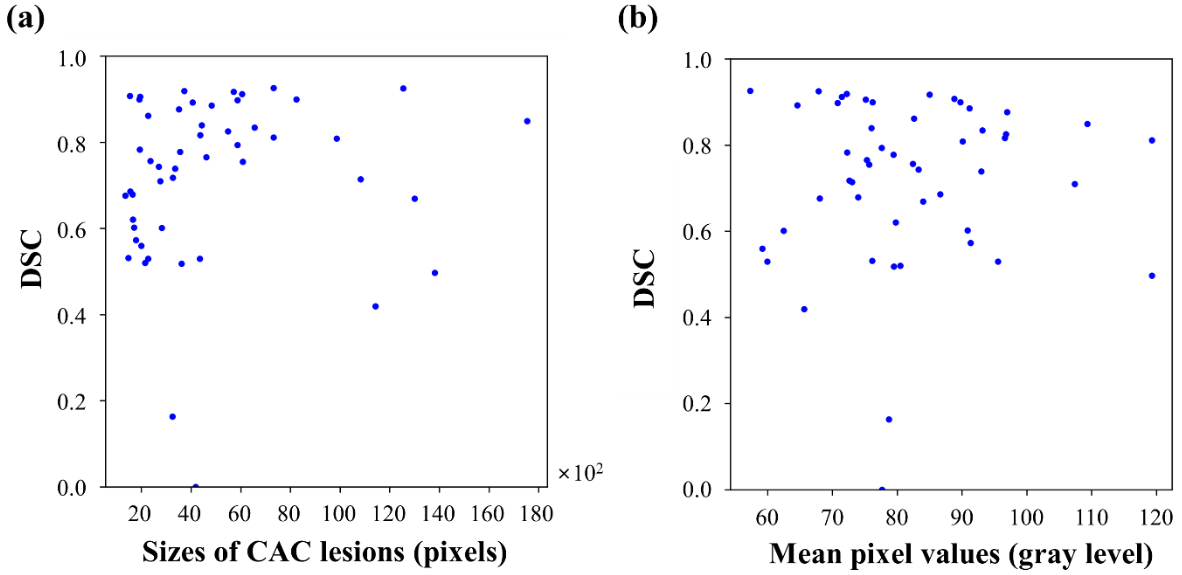

Figure S3. The dot plots for segmentation performance of CACSNet with EfficientNet-B4 (a) according to the sizes of CAC lesions, and (b) according to the mean pixel values of CAC lesions.
